# Evaluating the Safety and Performance of the KidneYou App for Chronic Kidney Disease: Protocol for an Italian Multicenter, Randomized, Open-Label, Premarket Study

**DOI:** 10.2196/75306

**Published:** 2026-02-19

**Authors:** Cesira Cafiero, Marco Gnesi, Anna Rita Maurizi, Andreana Foresta, Massimo Beccaria, Giorgia Campilongo, Marco Fiorentino, Paola Maria Acquaviva, Francesca Mastromauro, Marco Gorini, Loreto Gesualdo

**Affiliations:** 1 Nephrology, Dialysis and Transplantation Unit of the University Hospital Consortium, Bari, Italy; 2 Medical Evidence, Biopharmaceuticals Medical, AstraZeneca, Milan, Italy; 3 Medical Affairs, Biopharmaceuticals Medical, AstraZeneca, Milan, Italy; 4 Advice Pharma Group Srl, Milan, Italy; 5 Department of Precision and Regenerative Medicine and Ionian Area (DiMePre-J), Nephrology Dialysis and Transplantation Unit, University of Bari Aldo Moro, Piazza Umberto I, Bari, BA, 70121, Italy, 39 3204394546; 6 Medical Evidence EUCAN, Biopharmaceuticals Medical, AstraZeneca, Milan, Italy; 7 Innovation Unit & Business Excellence, AstraZeneca, Milan, Italy

**Keywords:** eHealth, digital therapeutics, mHealth, mobile health, lifestyle intervention., chronic kidney disease

## Abstract

**Background:**

Chronic kidney disease (CKD) is characterized by long-term structural or functional kidney abnormalities, often progressing over decades and potentially leading to kidney failure, treatable only by dialysis or transplantation. Nutritional programs (NPs), physical activity (PA) programs, and mindfulness programs (MPs) can play a key role in conservative CKD management, aiming to slow the progression of symptoms, decrease drug load, reduce stress, and delay dialysis. KidneYou (Advice Pharma Group Srl) integrates nutrition, exercise, and MPs to improve the health of patients with CKD by promoting lifestyle changes and stress reduction. As an innovative medical tool, KidneYou is designed to address an unmet need by providing a nonpharmacological approach for better managing CKD, empowering patients to manage their condition more effectively and sustain a healthier lifestyle, which could ultimately lead to improved disease outcomes.

**Objective:**

This study aims to evaluate the efficacy and safety of KidneYou, a digital medical device that delivers personalized NPs, PA programs, and MPs to patients with CKD, in comparison with the standard of care.

**Methods:**

This is a multicenter, open-label, randomized, parallel-arm trial aimed at evaluating health improvement in patients with CKD exposed to a nonpharmacological treatment involving an NP, PA program, and MP delivered to the patient by digital technology (investigational arm) or a standard approach (paper diary and control arm) for 3 months. The primary aim of the study is to evaluate the efficacy of KidneYou, defined by the achievement of at least 1 of the 3 conditions described in the composite primary end point: a reduction of at least 10% of azoturia (gram per 24 h), an increase of at least 15% of distance (meters in the 6-min walk test), or a decrease of at least 10% of perceived stress in CKD KidneYou app users compared to CKD KidneYou app nonusers after 3 months.

**Results:**

Data will be analyzed and presented in accordance with international CONSORT (Consolidated Standards of Reporting Trials) guidelines. Recruitment began in July 2022 and stopped at all sites in April 2024. Data analysis is currently ongoing. The results are expected to be published in early 2026.

**Conclusions:**

The results of this trial will guide further studies on the impact of digital devices to help patients with CKD in improving their condition more effectively and sustain a healthier lifestyle, which could ultimately lead to improved disease outcomes.

## Introduction

Chronic kidney disease (CKD) is a progressive condition defined by structural or functional abnormalities of the kidneys with health implications, persisting for over 3 months [1]. It is classified based on cause, glomerular filtration rate (GFR) category, and albuminuria category and includes various disorders that affect kidney structure and function, with diverse clinical presentations depending on the cause, severity, and progression rate. While some forms of CKD progress rapidly, most evolve over decades. Kidney failure, the most serious outcome, can only be treated with dialysis or transplantation [1]. Moreover, CKD is linked to an increased risk of acute kidney injury, which can worsen outcomes and accelerate CKD progression. Early identification and referral for specialized care can improve both clinical and economic outcomes [1].

Adherence to lifestyle interventions in patients with CKD presents persistent challenges, as patients often contend with physical, emotional, and cognitive barriers that impede consistent engagement with recommended dietary, exercise, and stress management routines. Digital health tools, particularly mobile apps, represent a promising solution to improve adherence by offering tailored, accessible support. With smartphone use increasingly widespread, these platforms can provide patients with CKD with real-time, personalized guidance, enhancing both engagement and self-management.

The nutritional program (NP) is an important component of the conservative management of patients with CKD, which precedes and integrates the pharmacological therapies. Generally, the objectives of NP are to maintain an optimal nutritional state, prevent or correct the signs, symptoms, and complications related to chronic renal insufficiency, and delay the start of dialysis. In addition, NP enables a reduction in the drug load (and related side effects and interactions) and can allow for safe and effective use of lower doses of dialysis, even when the GFR continues to diminish [2]. The aim of NP is to provide patients with CKD with an adequate caloric intake, control the sodium and potassium intake, and reduce phosphate intake. Beyond the quantitative aspects, dietary support also calls for modification in the quality of food, favoring foods of plant origin, which induce favorable effects on phosphate metabolism and acid-base balance, with better control of arterial pressure and renal hemodynamics [2,3]. Physical exercise in patients with CKD is another nonpharmacological tool aimed at improving health in this patient population. Despite large scientific evidence produced over the years about exercise tolerance and training among patients on dialysis, individuals with nondialysis-dependent CKD have been relatively understudied, perhaps because of the heterogeneity of the CKD population [4,5]. The clinical evidence produced for individuals with CKD is limited in both quality and quantity. Data from uncontrolled studies and small randomized controlled trials showed that exercise training results in improved physical performance and functioning. Of note, although the current literature does not allow for drawing definitive conclusions about the role of exercise training on the progression of CKD, no study has reported worsening of kidney function as a result of exercise training. Exercise appears to be safe in patients with CKD if begun at moderate intensity and increased gradually. The evidence suggests that the risk of remaining inactive is greater [6].

The mindfulness program (MP) aims to reduce stress perceived by patients with CKD and to improve their coping capacities in response to the psychological burden caused by living with an active chronic disease, the complexity of ongoing therapies, including concomitant ones, and the lifestyle changes required by dietary and exercise programs. Scientific evidence has shown that, when a stress-coping program based on mindfulness meditation is applied to stressed individuals, a significant reduction in perceived stress, anxiety, and depression is observed [7-9].

While the benefits of nutritional counseling, physical activity (PA) promotion, and behavioral support have been extensively demonstrated as stand-alone interventions, growing evidence suggests that integrating them in multicomponent lifestyle programs, particularly when supported by digital tools, may enhance patient engagement and lead to more sustained health improvements over time [10]. Despite the growing availability of digital tools, few interventions specifically designed for patients with CKD combine nutritional guidance, PA promotion, and stress management into a single, integrated platform [11].

The KidneYou app (Advice Pharma Group Srl) is an investigational medical device intended to administer these 3 nonpharmacological programs simultaneously to patients with CKD, to monitor patients’ performance and adherence during the study, and to improve their health status.

The aim of this study is to determine whether the investigational medical device KidneYou, randomly assigned to patients with CKD (G3b or G4 categories; A1 or A2 or A3 categories), can improve the CKD-related health conditions by positive changes in the patient’s lifestyle (ie, healthy eating and PA participation) and by means of cumulative stress reduction (ie, MP) originating from the underlying chronic disease and from diagnosis and treatment interventions necessary to manage it.

## Methods

### Study Intervention

KidneYou, an app for mobile devices developed by Advice Pharma Group Srl, on its electronic data capture (EDC) ICE technology, is an investigational medical device designed to deliver digital therapy (DT) to patients. DT, as part of digital health, uses high-quality digital technologies to stimulate lifestyle changes in patients.

A multidisciplinary board, composed of clinicians, nutritionists, psychiatrists, and graphic designers, has been established. The team worked collaboratively to map out the patient journey for individuals with CKD, identifying key stages within the disease management process. From this journey, critical touchpoints and challenges in CKD management were pointed out, emphasizing factors that play a pivotal role in improving patients’ physical and mental well-being.

Each specialist contributed to defining the core domains of the app. Nutritionists were involved in personalized NPs based on patients’ characteristics and needs, whereas psychiatrists contributed to the design of mindfulness practices tailored to address mental health challenges in patients with CKD. Physiatrists played a crucial role in developing the PA components, ensuring that exercise regimens were safe, effective, and adaptable to individuals with CKD.

Using these multidisciplinary inputs, a computational framework that generates a personalized algorithm was developed, incorporating customized nutrition, PA, and mindfulness strategies, all tailored to individual needs based on their specific health parameters.

To further enhance patient engagement, gamification elements were incorporated into the app design. These features were carefully crafted to encourage consistent app usage and promote a more interactive and enjoyable experience. By turning health management into an engaging activity, the app aims to foster long-term adherence to the prescribed treatment plan.

The overall design of the app reflects a patient-centered approach, combining clinical expertise from multiple fields with innovative digital solutions. This ensures that patients with CKD receive a comprehensive, holistic management experience that is both scientifically grounded and tailored to their specific needs.

**Figure 1.**
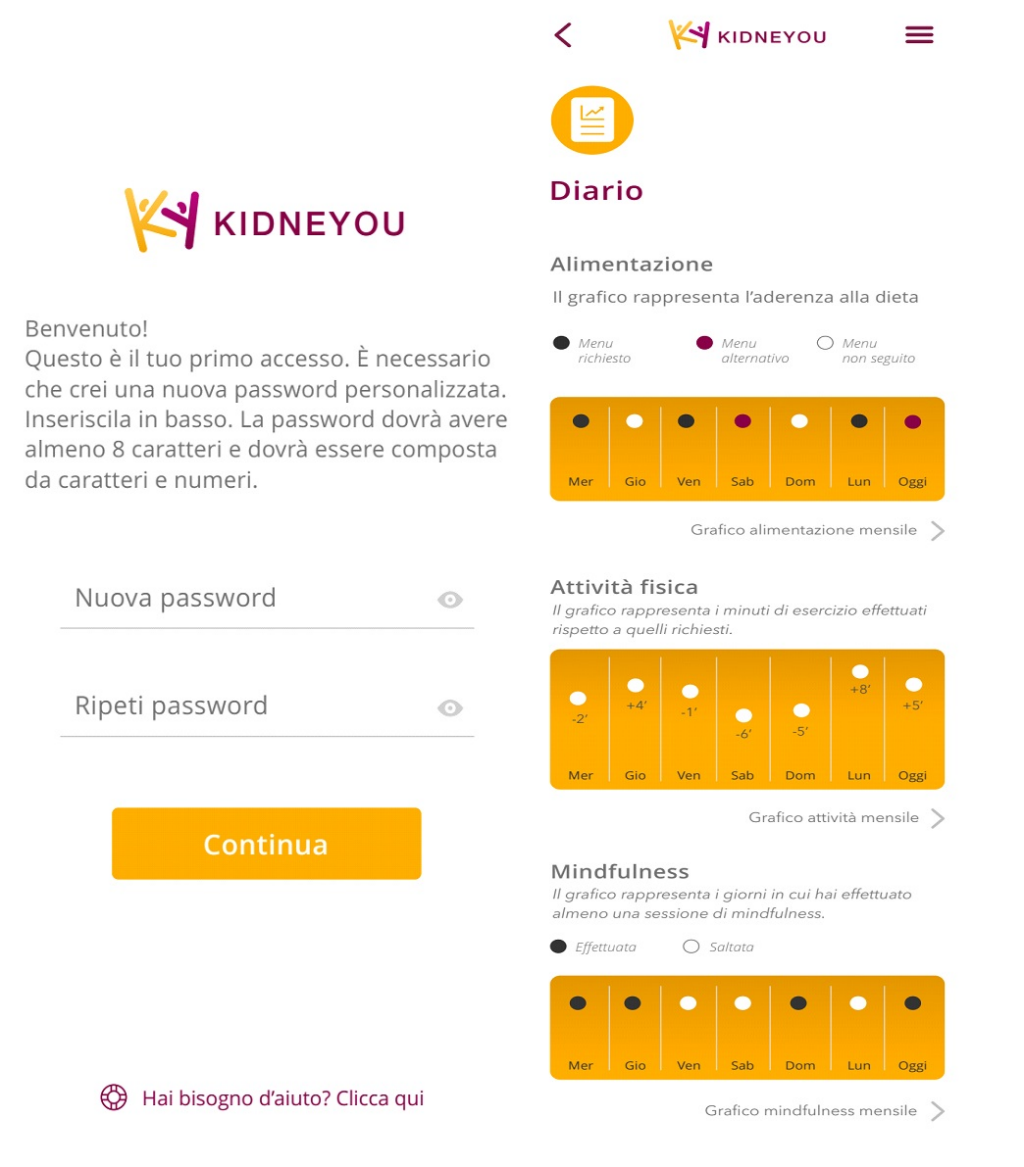
Login page of KidneYou (Advice Pharma Group Srl).

KidneYou has been designed to improve the commitment of patients in following clinical directives by working on three therapeutic areas: NPs, PAs, and MPs. All these programs are administered by means of DT for patients included in the intervention group. Images of KidneYou are presented in Figure 1.

The NP assigned by the app is based on gender, BMI, and estimated GFR (eGFR; values both measured at baseline) and is a customized low-protein dietary plan. The plan includes 5 daily meals, which vary every day of the week to maintain the proposed meals attractive for the patient and increase their compliance with the dietary plan. In addition, the NP proposes to each patient further daily alternatives to the first and second courses and side dishes to further augment food variability, reduce as much as possible dietetic monotony, and minimize abandonment or severe deviation from the assigned diet.

The PA assigned by the DT is based on an individual’s background fitness level and patient with CKD history, assessed at baseline. The exercise program is customized in terms of recommended type of PA, minutes of exercise per day, number of days per week, and level of intensity. At the end of each daily exercise, the patient selects the real number of minutes they spent exercising and rates the PA as too light, very good, or too heavy.

The MP aims to help patients follow stress-reducing activities; it consists of multimedia content (audio files) with different mindfulness meditation paths, allowing the patient to be guided by the therapist’s voice through different forms of exercise (eg, breath control, body scan, and virtual sound environments), all aimed at containing stress.

### Study Design

This is a multicenter, randomized, 2-arm, comparative, parallel, open-label, premarket study on individuals affected by CKD belonging to GFR categories G3b or G4, and with persistent albuminuria categories A1, A2, or A3 (A3 limited to >300 mg/g [>30 mg/mmol] and <3000 mg/g [<300 mg/mmol]).

This study is a premarket clinical investigation conducted in accordance with European Union Medical Device Regulation n.2017/745 requirements, with the goal of generating clinical evidence necessary for Conformité Européenne marking of the investigational medical device.

Enrolled participants are randomly allocated in a ratio of 1:1 to one of the two treatment arms using a randomization list that is computer generated by the trial statistician and provided through a web-based approach (via interactive response technology or randomization and trial supply management): group A (KidneYou users; investigational arm) or group B (KidneYou nonusers; control arm). A randomization list with random permuted blocks of different sizes (2 or 4 or 6 or 8) has been prepared and included in the electronic case report form (eCRF).

### Ethical Considerations

The study is conducted under the clinical investigation plan and international guidelines, including the most recent versions of the Declaration of Helsinki, the International Conference on Harmonization guidelines for Good Clinical Practice, European Union Medical Device Regulation 2017/745, and International Organization for Standardization (ISO) 14155, as well as all applicable local laws and regulations. The study was approved by the Territorial Ethics Committee (Comitato Etico Territoriale Regione Puglia of the Azienda Ospedaliero-Universitaria Consorziale Policlinico Di Bari, Institutional Ethics Committee protocol number 0036279, April 19, 2022) and by the Italian Ministry of Health (protocol number 2061; October 19, 2022). Informed consent is collected from all the participants involved in the study. No financial compensation was provided to the participants. All participant data are collected in a pseudonymized form and treated confidentially in compliance with applicable privacy laws, including the General Data Protection Regulation. Data are stored securely and used solely for research purposes.

The study team disseminates the trial results by making a summary publicly available on ClinicalTrials.gov, presenting findings at national and international conferences, and publishing them in peer-reviewed journals.

### Treatment Arms

In the intervention group (group A), the NP and PA program are administered through KidneYou, supplemented by guided stress-reduction activities.

In the control group (group B), the NP and PA program are administered by means of a paper diary. No indications are given to patients in this group regarding stress reduction activities.

### Duration of Study

For each patient, the treatment lasts for 3 consecutive months (12 wk), during which lifestyle changes are implemented with or without KidneYou. At the end of this period, the lifestyle program ends, followed by a follow-up visit 1 month later (at 16 wk).

### Study Setting

The trial involves 12 Italian clinical centers, all specialized in the treatment of renal diseases, distributed across different Italian regions, and provides a comprehensive representation of the country’s diverse lifestyles and dietary habits. A detailed list of the participating sites and their respective staff can be found in Multimedia Appendix 1.

### Study Population

The inclusion and exclusion criteria are presented in Textbox 1.

Textbox 1. **Inclusion and exclusion criteria.**

**Inclusion criteria**

- Age ≥18 years at the time of signing informed consent.
- Participants with chronic kidney disease classified as glomerular filtration rate (GFR) category G3b (30-44 mL/min/1.73 m²) or G4 (15-29 mL/min/1.73 m²); persistent albuminuria category A1 (<30 mg/g), A2 (30-300 mg/g), or A3 (>300 mg/g and <3000 mg/g).
- Presence of at least 1 causal etiology: diabetes, arterial hypertension, chronic glomerulonephritis, and cystic kidney diseases (eg, polycystic kidney, nephronophthisis, cortical or tubular glomerular cysts, renal medulla cystic diseases, and tuberous sclerosis).
- Abnormalities of kidney function (GFR and albuminuria) and structure (causal etiologies) present for >3 months with health implications.
- Perceived Stress Scale total score >12.
- Sex: male and female.
- Capability of giving informed consent, including compliance with requirements and restrictions.
- Ownership of a mobile phone, willingness to use mobile apps, and ability to download KidneYou.
- Native speakers of Italian or foreign individuals with a full understanding of Italian (all programs provided in Italian).

**Exclusion criteria**

- Medical conditions: (1) acute and chronic joint disease; (2) acute or chronic muscle diseases; (3) history or current depression; (4) sleep disturbance; (5) suicidal ideation or any mental disorder; (6) refusal or inability to follow dietary rules; (7) chewing disorder; (8) lack of motivation to follow nutritional program, physical activity, and psychological programs; (9) positive hepatitis C antibody, hepatitis B virus surface antigen or core antibody, Child-Pugh class C, and HIV positive; (10) drug or alcohol abuse; (11) QT-interval prolongation or congenital long QT-interval syndrome and history of arrhythmia; (12) cancer; and (13) autoimmune diseases, Addison disease, and amyloidosis.
- Prior or concomitant therapies and other exclusions: (1) any change, termination, addition, or replacement of medication in the current treatment strategy; (2) participation in another clinical study in the last 6 months; (3) involvement in the planning or conduct of the study; (4) investigator judgment that participation is not advisable; (5) previous enrollment or randomization in this study; (6) currently pregnant or breastfeeding (women only); and (7) any other condition making participation undesirable in the investigator’s opinion.

### Main Objectives and End Points

#### Primary Objective

The primary objective is (1) to improve the quality and quantity of dietary intake in patients using KidneYou compared to patients not provided with the app after 3 months of a customized NP, (2) to increase energy expenditure in patients using KidneYou compared to patients not provided with KidneYou after 3 months of a customized PA program, or (3) to relieve the stress related to the underlying CKD condition in patients using KidneYou compared to patients not using KidneYou after 3 months of study.

#### Secondary Objectives

The secondary objectives are as follows:

1. To help patients achieve 2 or more of the aforementioned objectives
2. To describe each primary objective achieved in each randomized group
3. To stratify the primary objective results by G3b and G4 categories of CKD classification
4. To stratify the primary objective results by A1, A2, and A3 (A3 limited to >300 mg/g [>30 mg/mmol] and <3000 mg/g [<300 mg/mmol]) category of CKD classification
5. To assess the degree of azoturia reduction (gram per 24 h) in each treatment group during the study
6. To assess differences in azoturia reduction (gram per 24 h) between the 2 randomized groups during the study
7. To assess the degree of physical function improvements in each treatment group during the 6-minute walk test (6-MWT)
8. To assess differences in physical function improvements between the 2 randomized groups during the study
9. To assess the degree of PA increase (min) in each treatment group during the study
10. To assess the degree of stress reduction (Perceived Stress Scale [PSS] of Cohen [12]) in each treatment group during the study
11. To assess the differences in stress reduction (PSS of Cohen) between the 2 randomized groups during the study
12. To detect the acceptability level among KidneYou users.

#### Safety Objective

The safety objective is to describe the safety profile in the 2 treatment groups.

#### Primary End Point

The primary end point is as follows: (1) to evaluate the achievement of a mean reduction of at least 10% of azoturia (gram per 24 h) in CKD KidneYou users exposed to a 3-month NP compared to CKD KidneYou nonusers exposed to the same 3-month NP, (2) to evaluate the achievement of a mean increase of at least 15% of the distance (meters in the 6-MWT) in CKD KidneYou users exposed to a 3-month PA program compared to CKD KidneYou nonusers exposed to the same 3-month PA program, or (3) to evaluate the achievement of a mean decrease of at least 10% of perceived stress (according to the PSS of Cohen) in CKD KidneYou users exposed to a 3-month MP compared to CKD KidneYou nonusers and not exposed to the same MP.

#### Secondary End Points

The secondary end points are as follows:

1. To evaluate the achievement of 2 or more of the aforementioned components of the primary end point
2. To assess the achievement of every single component of the primary end point in each randomized group
3. To stratify the within-group changes and the between-group differences in changes of primary end point results by the eGFR category of patients with CKD (eg, G3b and G4)
4. To stratify the within-group changes and the between-group differences in changes of primary end point results by the albuminuria category of patients with CKD (eg, A1, A2, and A3)
5. To assess the within-group changes of mean values of azoturia (gram per 24 h) in each treatment group during the study visits (ie, V0, V2, and V3)
6. To assess the between-group differences in changes of mean values of azoturia (gram per 24 h) during the study visits (ie, V0, V2, and V3)
7. To assess the within-group changes of mean values of heart rate (HR), blood pressure (mm Hg), and O_2_ saturation in each treatment group during the performance of the 6-MWTs
8. To assess the between-group differences in changes in mean values of HR, blood pressure (mm Hg), and O_2_ saturation during the performance of the 6-MWTs
9. To assess the within-group changes in mean values of PA (min) self-reported by patients in each treatment group during the study visits (ie, V0, V2, and V3)
10. To assess the within-group changes of mean values of stress reduction (according to the PSS of Cohen) in each treatment group during the study visits (ie, V0, V2, and V3)
11. To assess the between-group differences in changes of mean values of stress reduction (according to the PSS of Cohen) during the study visits (ie, V0, V2, and V3)
12. To assess patient satisfaction with the KidneYou App by using the Net Promoter Score system. With a score ≥7, the participant will be considered a promoter of the app

#### Safety End Point

The safety end point is to assess the rate of adverse events (AEs), adverse device effects, and abnormality of laboratory parameters between the 2 treatment groups across the study (ie, V1, V2, and V3).

#### Tertiary or Exploratory End Points

The tertiary or exploratory end points are as follows:

1. To assess the between-group differences in changes of mean values of PA (min) self-reported by patients during the study visits (ie, V0, V2, and V3)
2. To assess the within-group changes and the between-group differences in changes of urinary levels of phosphorus, calcium, potassium, sodium, chlorine, bicarbonate, albumin, creatinine, glucose, and uric acid by urine samples during the study visits (ie, V0, V2, and V3)
3. To assess the within-group changes and the between-group differences in changes of urinary levels of other compounds (ie, density, pH, proteins, ketones, hemoglobin, bilirubin, nitrites, white cells, red blood cells, casts, crystals, and epithelial cells) by urine samples during the study visits (ie, V0, V2, and V3)
4. To assess the within-group changes and the between-group differences of changes in blood levels of electrolytes (potassium, sodium, chloride, and bicarbonate), minerals (phosphorus and calcium), proteins (albumin), metabolic products (nitrogen and creatinine), sugar (glucose), and uric acid in the blood samples during the study visits (ie, V0, V2, and V3)
5. To assess the within-group changes and the between-group differences of changes of vital signs (systolic blood pressure or diastolic blood pressure, HR, and respiratory rate) during the study visits (ie, V0, V1, V2, and V3)
6. To assess the between-group differences of changes in mean values of impedancemetry (lean mass and fat mass) during the study visits (ie, V0, V2, and V3)
7. To assess the within-group changes and the between-group differences of changes of signs and symptoms characterizing CKD during the study visits (ie, V0, V1, V2, and V3)
8. To assess the within-group compliance (adherence) by measuring the overall time (min) of KidneYou use during the 3 months of the study
9. To assess a possible correlation between levels of compliance with KidneYou use and the rate of achievement of the primary end point
10. To assess a possible correlation between levels of compliance (min) with KidneYou and the customer experience rate according to Net Promoter Score

### Assessments

Each participant undergoes 4 visits and is examined according to the following schedule: baseline (V0), 6-week visit (V1), 12-week visit (V2), and 4-week follow-up visit (V3). During the baseline visit, the patient signs the informed consent form and is evaluated for inclusion and exclusion criteria. Once enrollment is confirmed, the patient is randomized. The PSS questionnaire is administered to all enrolled patients, regardless of the randomization group. At V0, V1, V2, and V3, a complete physical examination is performed; vital signs (systolic blood pressure, diastolic blood pressure, HR, and respiratory rate), ongoing medications for CKD, and concomitant medications are collected.

At V0, V2, and V3, laboratory tests are also performed and include hematology (complete blood count, glycemia, proteins [albumin], and creatinine), minerals (phosphorus and calcium), electrolytes (sodium, potassium, chloride, and bicarbonate), azotemia, and uric acid; lipid profile, that is, total cholesterol, high-density lipoprotein cholesterol, low-density lipoprotein cholesterol, and triglycerides; and urine tests (24 h urinary excretion collection), that is, urea nitrogen, uric acid, minerals (phosphorus and calcium), electrolytes (potassium, sodium, chloride, and bicarbonate), albumin, creatinine, glucose, density, pH, proteins, ketones, hemoglobin, bilirubin, nitrites, white cells, red blood cells, casts, crystals, epithelial cells, and serum pregnancy test, only for women of childbearing potential.

All patients undergo the 6-MWT and bioimpedance test. Safety, including the appearance of adverse device effects and abnormalities in physical and laboratory parameters, is assessed throughout the entire study period.

### Data Collection and Management

The KidneYou software consists of 3 different components.

The mobile app enables the patient to receive dietary indications, exercise categories, and mindfulness content and to enter information related to the activities performed. It also allows the patient to provide feedback through questions and evaluations on the activities performed.

The ICE Platform, which can be accessed through a dedicated control panel after authentication and authorization, allows the physician to enter patient data into the platform. The platform also displays data on patient adherence to recommended activities.

The backend is the part of the software dedicated to exchanging communications between the mobile app and the ICE platform. It receives patient input from the app, including self-reported adherence to activities, which is sent to the platform for visualization.

The KidneYou database has been developed using EDC technology (ICE, Advice Pharma), compliant with the regulations for clinical data management to facilitate the study data analysis.

Data are collected for each participant in eCRFs. The eCRF is designed by Advice Pharma Group using the ICE software.

ICE (version 2.6.2) is the EDC solution provided by Advice Pharma. This system is a fully web-based Integrated Development Environment designed to generate customizable eCRFs.

The EDC system consists of the database application and integrated tools for query management and audit trail. Data are reviewed and checked for completeness and validity before, during, and after data entry. The data cleaning process includes different levels of control: source data verification and quality checks at the data entry level, automatic controls on the ICE database, and database data review performed by the data manager, with the production of manual eQueries.

Entered data are subject to visual and electronic validation by data management and monitoring staff. Any errors, inconsistencies, and unexpected values in the data are noted and queried with the site staff for resolution.

The data manager reviews data for each patient upon study withdrawal, after all data are entered, and after source data verification is completed. Data cleaning continues until all discrepancies are resolved. Data changes resulting from queries are made by the investigator directly in the clinical database.

Concomitant medications are coded using the latest available version of the *World Health Organization Drug Dictionary Enhanced* (WHO DDE). The data manager performs the coding centrally using the dedicated ICE coding tool, matching the term entered by the investigator with the appropriate term in the relevant dictionary.

Medical coding is performed at the end of the study before database lock.

### Statistics

#### Sample Size Calculation

This study is designed to test the null hypothesis that the percentage *success* (defined by the achievement of at least one of the 3 conditions described in the primary end point, with no hierarchical structure among them) in KidneYou nonusers is equal to the percentage “success” in KidneYou users. The alternative hypothesis is that the percentage “success” in KidneYou nonusers is different from the percentage “success” in KidneYou users.

The sample size is estimated to assess the study hypothesis on the primary end point.

The sample size calculation is based on the combination of the expected proportion of success in the control group and the expected delta (Δ) of success in the experimental group.

With a significance level α equal to .05 and a power equal to 80%, a sample size of 190 participants (95 per group) is required to detect as significant, if any, a success delta in favor of the experimental group equal to 20% (under the hypothesis of success rate of 40% in the control group and success rate of 60% in the experimental group). Considering a dropout rate of approximately 10%, a total of 210 patients (105 per group) will have to be enrolled. A competitive enrollment approach across the centers involved in the study will be used to reach the sample size efficiently.

#### Statistical Analysis

All the analyses on primary, secondary, and tertiary or exploratory end points will be carried out in the intention-to-treat population—as main analyses—and in per protocol population, as sensitivity analyses. Safety end points will be described only for the per protocol population.

Data will be analyzed and presented in accordance with the international CONSORT (Consolidated Standards of Reporting Trials) guidelines and analyzed according to the intention-to-treat principle.

Data analyses will be conducted using SAS (version 9.4; SAS Institute) statistical software.

#### Primary Analysis on Primary End Point

The primary efficacy end point is the percentage of achievement of a mean reduction of at least 10% of azoturia (grams per 24 h) or the achievement of a mean increase of at least 15% of distance (meters in the 6-MWT) or the achievement of a mean decrease of at least 10% of perceived stress (according to the PSS of Cohen) in KidneYou users exposed to a 3-month NP, PA program, and MP compared to KidneYou nonusers and not exposed to the same 3-month programs.

The observed percentages (in KidneYou users vs KidneYou nonusers) will be tested using a chi-square test or the Fisher exact test, as appropriate.

The secondary analyses on the primary end point include (1) achievement of each single component of the primary end point in each randomized group, (2) achievement of 2 or more of the aforementioned components of the primary end point, and (3) assessment of between-group differences in primary end point results by estimated eGFR category of patients with CKD (eg, G3b and G4) and by albuminuria category of patients with CKD (eg, A1, A2, and A3).

#### Secondary End Points

The secondary end points include the evaluation of many parameters (both continuous and categorical) through comparisons both between groups and within groups.

Between-group differences will be investigated with 2-tailed unpaired *t* tests (or the corresponding nonparametric Wilcoxon-Mann-Whitney tests, as appropriate) in case of a continuous variable and with chi-square test or the Fisher exact test in case of a categorical variable.

Within-group differences will be investigated with paired *t* tests (or the corresponding nonparametric Wilcoxon-Mann-Whitney tests, as appropriate) in case of a continuous variable, with the McNemar test in cases of a categorical one.

If appropriate, the percentage change with respect to the baseline value will be calculated for each interesting time point.

Global approaches, such as mixed linear models for repeated measures, will also be considered.

#### Tertiary or Exploratory End Points

Tertiary or exploratory end points that involve a comparison between and within groups will be analyzed as described in the “Secondary End Points” section.

To assess possible correlation between levels of compliance to KidneYou use and other parameters of interest, the most appropriate test will be used. In particular, in case of continuous or numerical parameters, the Pearson correlation coefficient (or Spearman, as appropriate) will be computed; in case of discrete or dichotomous parameters, the association will be assessed with 2-tailed unpaired *t* tests or the corresponding nonparametric Wilcoxon-Mann-Whitney tests, as appropriate.

#### Safety

Medical AEs, medical device AEs, and laboratory parameter abnormalities will be collected during the study.

Medical AEs will be coded to a preferred term and primary system organ class, using the current version of the *Medical Dictionary for Regulatory Activities* (MedDRA; International Council for Harmonisation of Technical Requirements for Pharmaceuticals for Human Use) dictionary. AEs will be tabulated as preferred term, system organ class, and frequency for the safety population.

The statistical difference in the rate of AEs, device AEs, and laboratory parameter abnormalities will be assessed through the chi-square test or the Fisher exact test.

## Results

Data will be analyzed and presented following the guidelines outlined in the international CONSORT to ensure the integrity and transparency of the results. The primary and secondary end points will be rigorously examined to assess the effectiveness of the KidneYou app in promoting adherence to nonpharmacological interventions (such as NP, PA program, and MPs) and improving health outcomes in patients with CKD.

Recruitment began in July 2022 and was completed in April 2024 across 12 Italian nephrology centers with a total number of 210 participants enrolled. Data analysis is currently ongoing. The results are expected to be published by early 2026.

## Discussion

### Anticipated Findings

This study is designed to evaluate the anticipated performance of KidneYou, a digital therapeutic tool delivering personalized NP, PA program, and MPs, which can improve adherence to lifestyle changes and health outcomes in patients with CKD, compared to the standard approach.

If effective, the KidneYou model could guide future digital health solutions for CKD and other chronic diseases, offering a scalable, nonpharmacological approach. These solutions may complement medical treatments, reduce costs, and enhance patient adherence to lifestyle changes. By providing an accessible, patient-centered tool, this approach could improve treatment outcomes and help patients with CKD manage their condition more effectively.

### Comparison With Prior Work

Previous digital interventions for patients with CKD have largely focused on single domains such as nutritional guidance or physical activity, with limited evidence on integration across multiple lifestyle components. To the best of our knowledge, no existing digital solution for CKD has combined all 3 nonpharmacological domains in a single, personalized platform. This trial is designed to fill that gap.

### Strengths and Limitations

The strengths of this study include its multicenter design, the use of a composite primary end point, and the personalization of interventions to individual patient needs. Limitations include the relatively short intervention period, the absence of a prespecified plan to disentangle the independent contribution of engagement features to adherence or outcomes, and the exclusion of patients with mental health conditions, such as depression or sleep disturbances, which are relatively common among individuals with CKD who could also benefit from mindfulness-based interventions. Nevertheless, this approach was necessary to standardize the study population and minimize potential confounding factors in this interventional trial. We also acknowledge that restricting participation to Italian speakers and tailoring the content to the Italian context may limit the international applicability of the findings. However, this design was necessary because the KidneYou App, including all its content, was specifically developed for Italian patients and is culturally, linguistically, and in terms of dietary content, tailored to Italy. Future studies may explore the adaptation of the app for other countries and languages to assess generalizability.

### Future Directions 

If the intervention proves to be effective, future research should investigate long-term outcomes and improve mechanisms of engagement. Studies could also explore optimization of individual program components and adaptation of the model for populations with other chronic diseases.

### Dissemination

The results of this study will be made publicly available on ClinicalTrials.gov and disseminated through peer-reviewed publications and presentations at national and international conferences, ensuring accessibility to both the scientific community and clinical stakeholders.

### Monitoring

The study is monitored in accordance with Good Clinical Practice guidelines and the study-specific monitoring plan. On-site monitoring visits are conducted to ensure protocol adherence, data accuracy, and participant safety. Source data verification is performed as per the monitoring plan. AEs and serious adverse events are assessed and reported in compliance with regulatory requirements.

## Supplementary material

10.2196/75306 Multimedia Appendix 1 List of study sites participating in the trial and corresponding principal investigators.

## Abbreviations

6-MWT: 6-minute walk test
AE: adverse event
CKD: chronic kidney disease
DT: digital therapy
eCRF: electronic case report form
EDC: electronic data capture
eGFR: estimated glomerular filtration rate
GFR: glomerular filtration rate
HR: heart rate
ISO: International Organization for Standardization
MedDRA: *Medical Dictionary for Regulatory Activities*
MP: mindfulness program
NP: nutritional program
PA: physical activity
PSS: Perceived Stress Scale
WHO DDE: *World Health Organization Drug Dictionary Enhanced*
