# Supplementary material for: Evaluating the Safety and Performance of the KidneYou App for Chronic Kidney Disease: Protocol for an Italian Multicenter, Randomized, Open-Label, Premarket Study
Source: JMIR Res Protoc. 2026 Feb 19;15:e75306. doi: 10.2196/75306 (PMC12933345; doi:10.2196/75306)
Supplement: Multimedia Appendix 1 [file resprot-v15-e75306-s001.docx]

**Title**: Rationale and design of an Italian multicenter, randomized, open-label, pre-market study to evaluate safety and performance of the innovative KidneYou app, in improving health of patients with chronic kidney disease by enhancing adherence to dietary, exercise and mindfulness programs.

**Supplementary Material**

**Sites list:**

1. Policlinico di Bari Azienda Ospedaliera Universitaria, PI: Prof. Loreto Gesualdo
2. Policlinico di Sant'Orsola, PI: Prof. Gaetano La Manna
3. Ospedale di Palmanova, PI: Dr. Massimiliano Tosto
4. Ospedale San Giovanni di Dio – (Osp. Torregalli), PI: Dr. Alberto Rosati
5. Ospedale della Murgia Fabio Perinei, PI: Dr. Giosafatte Pallotta
6. Ospedale Ecclesiastico Miulli, PI: Dr. Vincenzo Montinaro
7. Istituto Maugeri IRCCS Pavia, PI: Dr. Ciro Esposito
8. Azienda Ospedaliera Universitaria Federico II di Napoli, PI: Prof. Antonio Pisani
9. Ospedale San Bassiano, PI: Dr. Paolo Lentini
10. Ospedale Belcolle di Viterbo, PI: Dr.ssa Micol Manzuoli
11. Azienda Ospedaliera "G. Brotzu", PI: Dr. Antonello Pani
12. Ospedali Riuniti Torrette di Ancona, PI: Dr. Andrea Ranghino
